# Impact of the change from the seventh to eighth edition of the AJCC TNM classification of malignant tumours and comparison with the MACIS prognostic scoring system in non‐medullary thyroid cancer

**DOI:** 10.1002/bjs5.50182

**Published:** 2019-06-28

**Authors:** S. Dwamena, N. Patel, R. Egan, M. Stechman, D. Scott‐Coombes

**Affiliations:** ^1^ Department of Endocrine Surgery University Hospital of Wales Heath Park Cardiff CF14 4XW UK

## Abstract

**Background:**

In 2018, AJCC TNM staging changed for differentiated (DTC) and anaplastic (ATC) thyroid carcinoma. The impact of this change on mortality rates was investigated and compared with the MACIS prognostic score.

**Methods:**

Analysis of a prospective database of DTC/ATC was undertaken. Patients were staged according to TNM7 and TNM8 criteria, and MACIS scores calculated. Five‐year disease‐specific mortality rates were determined. Proportions were compared with Fisher's exact and χ^2^ goodness‐of‐fit tests.

**Results:**

Between August 2002 and December 2016, 310 patients had primary surgery for thyroid cancer. After exclusions, 159 patients (154 DTC, 5 ATC) remained to be studied. The MACIS score was less than 6 in 105 patients (66·0 per cent), 6–6·99 in 19 (11·9 per cent), 7–7·99 in 14 (8·8 per cent) and 8 or more in 21 (13·2 per cent), with corresponding disease‐specific 5‐year mortality rates of 0, 5, 14 and 86 per cent. For TNM7 the distribution was stage I in 53·5 per cent (85 patients), stage II in 10·1 per cent (16), stage III in 14·5 per cent (23) and stage IV in 22·0 per cent (35), and differed from that for TNM8: 76·7 per cent (122), 10·7 per cent (17), 4·4 per cent (7) and 8·2 per cent (13) respectively (*P* < 0·001). Overall disease‐specific 5‐year mortality rates by stage for TNM7 *versus* TNM8 were: stage I, 0 of 85 *versus* 3 of 100 (*P* = 0·251); stage II, 0 of 16 *versus* 6 of 16 (*P* = 0·018); stage III, 3 of 23 *versus* 2 of 7 (*P* = 0·565); stage IV, 20 of 32 *versus* 11 of 11 (*P* = 0·020).

**Conclusion:**

Compared with TNM7, TNM8 downstaged more patients to stage I and accurately reflected worse prognosis for stage IV disease. TNM8 is an inferior predictor of mortality compared with MACIS.

## Introduction

Staging systems for cancer divide patients into groups to predict prognosis and define treatment. The division of patients into low‐, intermediate‐ and high‐risk groups is important for personalized decision‐making, based on long‐term follow‐up and survival differences from population studies^1^. The TNM staging system is that recommended by the American Thyroid Association (ATA)^2^ and the British Thyroid Association^3^.

A key aspect of the TNM system is that there is an age cut‐off point that separates younger patients, who can only have stage I or II disease, from older patients, who can have stage I–IV. In previous editions of the TNM system, this division by age resulted in a poor correlation between risk of death and stage: patients with stage II disease could have a low, intermediate or even high risk of death^4^. In addition, in the seventh edition of the TNM classification (TNM7), patients aged over 45 years who had N1 status were upstaged to stage III, with a resultant increase in the administration of adjuvant radioiodine therapy^2^.

On 1 January 2018, the AJCC TNM7 classification was updated to the eighth edition (TNM8)^5^. For non‐medullary thyroid carcinoma, differentiated (DTC) and anaplastic (ATC), there were a number of key changes: an increase in the age used for stage I and II disease only from 45 to 55 years; T2 tumours were moved from stage II to stage I; and N1 status was moved from stage III to stage II disease. Minor thyroid extension detected only on histological examination was removed as a category, and a new category of T3a (for tumours larger than 4 cm and confined to the thyroid) was defined. T3b was defined as a new category for gross extension into strap muscles, and involvement of level VII nodes was moved from category N1b to N1a. Finally, TNM8 re‐emphasized the critical importance of gross extrathyroid extension.

In 1993, the MACIS (Metastases, Age, Completeness of resection, Invasion, Size) prognostic system for papillary thyroid cancer (PTC)^6^ was designed at the Mayo Clinic (Rochester, Minnesota, USA) (*Table* 1). This tool has a built‐in converter for age stratification (40 years or older), which results in a very accurate correlation between the prognostic score and risk of death^7^. It also disregards node positivity in the calculation of risk of dying, as this is a marker for recurrence rather than disease‐specific mortality.

**Table 1:** MACIS prognostic scoring table^5^

	Score
**Distant metastasis (spread of cancer to areas outside neck**)	
Yes	3
No	0
**Age when tumour discovered (years)**	
≤ 39	3·1
≥ 40	Age × 0·08
I**nvasion into surrounding areas of neck as seen by naked eye**	
Yes	1
No	0
**Complete resection (or removal) of tumour**	
No	1
Yes	0
**Size of tumour (cm)**	Size × 0·3

This study investigated the impact of the change in TNM staging in a cohort of patients, and compared outcomes with the long‐established MACIS prognostic scoring system.

## Methods

Analysis of a database of consecutive patients with DTC or ATC treated at a university teaching hospital was undertaken; this included all non‐medullary thyroid cancer (poorly differentiated, insular and anaplastic). Basic demographic data were collected for each individual analysed. Patients were staged or restaged, according to TNM7 and TNM8 criteria, and MACIS scores were calculated for each patient. Histopathology reports for individual patients were reviewed on an electronic clinical portal system.

### Statistical analysis

Stage‐by‐stage survival was compared between the two TNM classifications using Kaplan–Meier curves and log rank analysis. In addition, 5‐year disease‐specific mortality rates were calculated. Proportions were compared with Fisher's exact and χ^2^ tests. Analysis was undertaken using the software package SPSS® version 23 (IBM, Armonk, New York, USA).

## Results

Between August 2002 and December 2016, 310 patients had surgery for newly presenting thyroid cancer. Patients with medullary, squamous, metastatic and sarcoma lesions (51) and those with follow‐up of less than 36 months (100) were excluded, leaving 159 patients (154 DTC, 5 ATC) to be studied. There were no patients with non‐invasive follicular thyroid neoplasm with papillary‐like nuclear features in the cohort. Patients with ATC underwent total thyroidectomy (2), hemithyroidectomy (1) and exploration/biopsy alone (2). Of the remaining 154 patients, 142 (92·2 per cent) had a total thyroidectomy. Radioiodine ablation was undertaken in 128 patients (83·1 per cent) with non‐ATC DTC, most of whom had papillary carcinoma. Two patients with ATC received adjuvant chemoradiotherapy.

**Figure 1:**
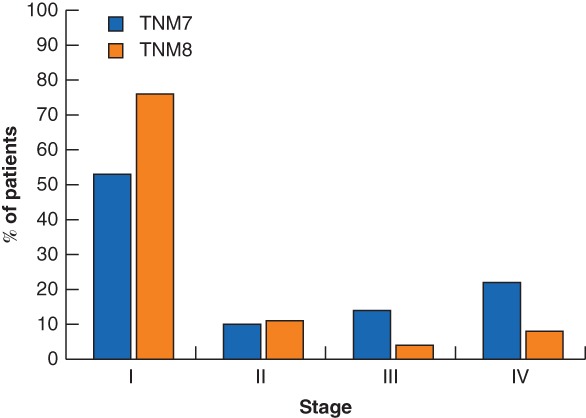
Stage distribution for the seventh (TNM7) and eighth (TNM8) editions of the AJCC TNM classification of malignant tumours

For TNM7 the distribution of stages was 53·5 per cent (85 of 159 patients) stage I, 10·1 per cent (16) stage II, 14·5 per cent (23) stage III and 22·0 per cent (35) stage IV, and differed from TNM8 where the distribution was 76·7 (122 patients) stage I, 10·7 per cent (17) stage II, 4·4 per cent (7) stage III and 8·2 per cent (13) stage IV (*P* < 0·001) (*Fig*. 1).

**Figure 2:**
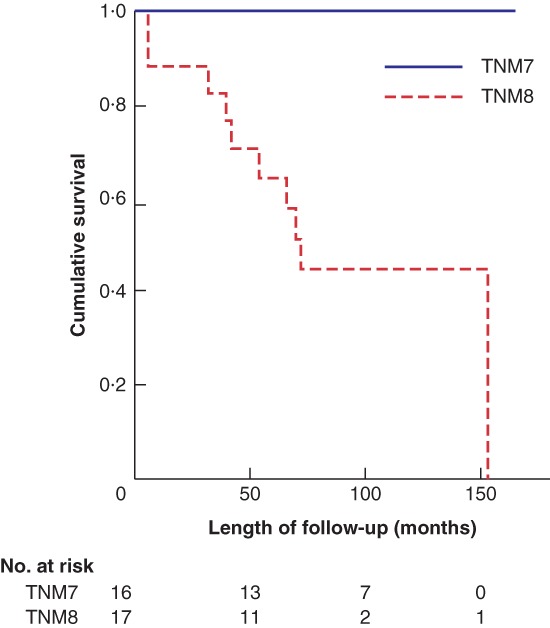
Kaplan–Meier analysis of overall survival in patients with stage II thyroid carcinoma classified according to TNM7 and TNM8 *P* = 0·005 (log rank test).

**Figure 3:**
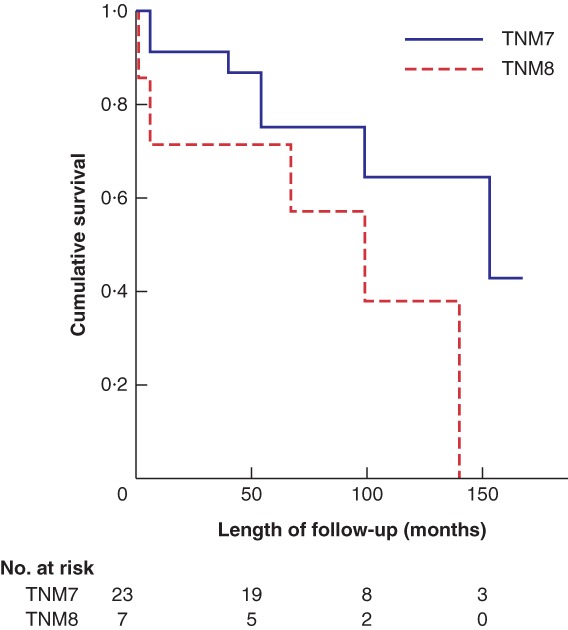
Kaplan–Meier analysis of overall survival in patients with stage III thyroid carcinoma classified according to TNM7 and TNM8 *P* = 0·104 (log rank test).

**Figure 4:**
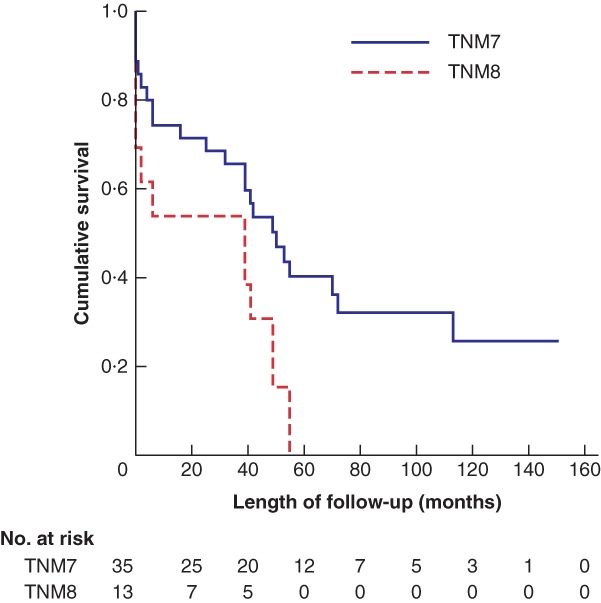
Kaplan–Meier analysis of overall survival in patients with stage IV thyroid carcinoma classified according to TNM7 and TNM8 *P* = 0·003 (log rank test).

Kaplan–Meier survival analysis demonstrated no differences in overall survival (OS) (*P* = 0·328) or disease‐free survival (DFS) (*P* = 0·353) for stage I disease, when comparing TNM7 and TNM8. The equivalent calculations for stage II demonstrated improved OS (*P* = 0·005) (*Fig*. 2) and DFS (*P* = 0·001) for patients staged using TNM7. OS (*P* = 0·104) (*Fig*. 3) and DSF (*P* = 0·213) were similar for patients with stage III disease, but for stage IV OS (*P* = 0·003) (*Fig*. 4) and DFS (*P* = 0·018) were again improved for patients staged with TNM7.

Overall disease‐specific 5‐year mortality rates by stage for TNM7 *versus* TNM8 were: stage I, 0 of 85 (0 per cent) *versus* three of 100 (3·0 per cent) (*P* = 0·251); stage II, 0 of 16 (0 per cent) *versus* six of 16 (38 per cent) (*P* = 0·018); stage III, three of 23 (13 per cent) *versus* two of seven (29 per cent) (*P* = 0·565); stage IV, 20 of 32 (63 per cent) *versus* 11 of 11 (100 per cent) (*P* = 0·020).

**Figure 5:**
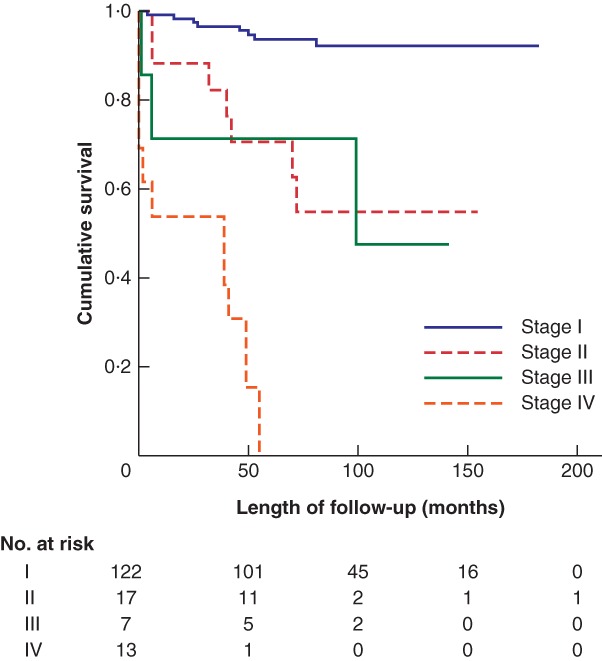
Kaplan–Meier analysis of overall survival in patients with stage I, II, III and IV disease classified according to TNM8 *P* = < 0.0001 (log rank test).

When considering survival using TNM8, the outcomes for stage II and III disease overlapped (*Fig*. 5).

**Figure 6:**
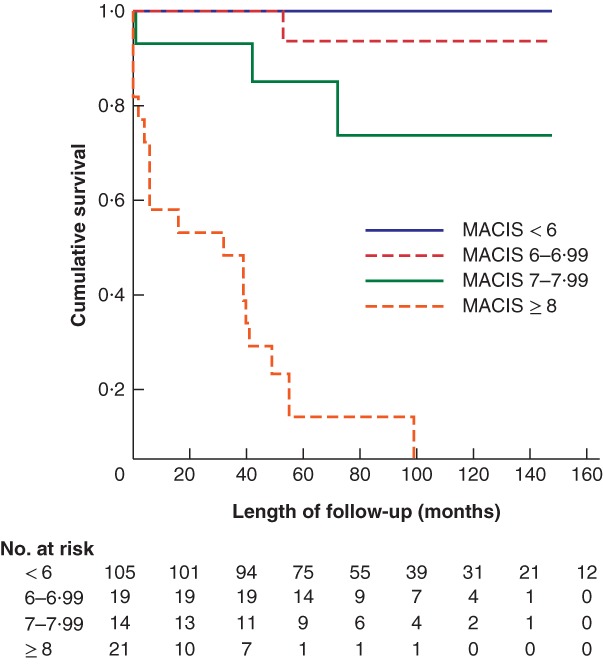
Kaplan–Meier analysis of overall survival in patients staged according to the MACIS staging system *P* = < 0.0001 (log rank test).

The MACIS score was less than 6 in 105 patients (66·0 per cent), 6–6·99 in 19 (11·9 per cent), 7–7·99 in 14 (8·8 per cent) and 8 or more in 21 (13·2 per cent), with corresponding disease‐specific 5‐year mortality rates of 0, 5, 14 and 86 per cent (*Fig*. 6).

## Discussion

The principal finding of this paper is that although the change from TNM7 to TNM8 downstaged many patients to stage I, it failed to spread out the distribution of mortality between the stages. Conversely, MACIS delivers an excellent correlation between risk groups and mortality.

Clinical management of patients with thyroid cancer is guided by assessment of both the risk of dying and the risk of disease recurrence. For recurrence, the ATA risk stratification system is recommended^8^. The emphasis of this paper is on risk of death and the TNM update. The principal consequences of the change from TNM7 to TNM8 have led to the downstaging of a significant number of patients to lower stages that more accurately reflect their risk of death from thyroid cancer^9^. The results of this study demonstrate that this change results in the downstaging of more patients to stage I disease. It also reflects more accurately the worse prognosis of patients with stage IV disease. However, the change in cut‐off point in age from 45 to 55 years has not smoothed out the distribution of mortality across stages II and III. TNM8 has introduced a gradation that infers a significant survival difference between stage I and II disease, and a universally poor prognosis for those with stage IV disease. Stage III disease, by contrast, is uncommon in TNM8 and appears to confer a mortality risk similar to that of stage II disease rather than being intermediate between the latter and grade IV disease.

Over the years there have been many prognostic scoring systems for thyroid cancer that have many common features including patient factors (age, sex) and tumour factors (size of primary tumour, local invasion and metastasis)^6^, ^7^, ^10^, ^11^, ^12^, ^13^, ^14^, ^15^, ^16^, ^17^, ^18^, ^19^, ^20^, ^21^, ^22^, ^23^, ^24^. Attempts have been made to compare these systems and identify those that provide the best information. Using the statistical model Proportion of Variation in survival time Explained^25^, some^26^ found no difference between the systems, and others^27^, ^28^ found that MACIS, TNM and EORTC systems were the most accurate, with MACIS the best overall for predicting cancer‐specific survival. In fact, the Austrian analysis^28^ found MACIS to be the pre‐eminent prognostic scoring system for PTC, but recommended that TNM would be adopted more readily owing to its widespread use in cancer prognosis and registries. This was endorsed by the ATA guidelines published in 2009^29^ and 2016^2^, and by the British Thyroid Association^3^.

The change from TNM7 to TNM8 sets out to address concerns about the mismatch between risk group and mortality rates. The change in the cut‐off point for stage II in TNM8 is a recognition that older age at presentation is associated with worsening prognosis^30^, but it is acknowledged that a single cut‐off point for age is likely to perform less well than models that consider age as a continuous variable (for example MACIS)^6^, ^9^. In developing a risk‐adapted approach for the management of thyroid cancer, Tuttle and colleagues^4^ pointed out that incomplete (macroscopic) excision of the primary tumour places the patient immediately into a high‐risk group. Incomplete excision has not been part of the TNM staging system until now, whereas MACIS is the only system that has always considered the presence of gross residual disease after the primary surgical resection^26^.

The results of the present study demonstrate that MACIS risk groups correlate with the risk of death and compare well with the original work on MACIS^6^. In this study, MACIS distributed outcomes within risk groups better than both TNM7 and TNM8, in all likelihood because it attributes increasing risk to increasing age and gives additional weighting for the presence of residual macroscopic disease at surgery. MACIS has been in existence for over 20 years and can readily be calculated (https://www.thyroid.org/professionals/calculators/thyroid‐cancer‐staging‐calculator/) from existing data sets. In contrast, TNM8 has newly defined categories that will require retrospective review of histology reports to convert previous TNM stages to TNM8, making comparison with historical data a burden. Although MACIS was designed for PTC, its predecessor (AGES – Age, tumour Grade, Extent and Size)^6^ has also been applied to follicular thyroid carcinoma^31^. It does appear that scoring systems derived for patients with PTC are predictive of outcome in follicular thyroid cancer^32^.

The reproducibility of MACIS and its ability to be applied in retrospect make it an attractive option for staging of non‐medullary thyroid carcinoma, either as the standard staging system or for use in conjunction with TNM.

## Disclosure

The authors declare no conflict of interest.
